# Pathology, Diagnosis, and Management of Sarcoma

**DOI:** 10.3390/ijms25126609

**Published:** 2024-06-15

**Authors:** Shinji Miwa, Katsuhiro Hayashi, Satoru Demura

**Affiliations:** Department of Orthopaedic Surgery, Graduate School of Medical Sciences, Kanazawa University, Kanazawa 920-8640, Japan

Due to the rarity and heterogeneity of sarcoma, investigation into molecular targets and new treatments has been particularly challenging. Although intensive chemotherapy and the establishment of surgical procedures have improved the outcomes of patients with sarcomas, the curative rate of recurrent and metastatic sarcomas remains unsatisfactory. Recent basic studies have revealed some of the mechanisms of the progression and metastasis of malignancies, including proliferation, apoptosis, angiogenesis, tumor microenvironment, migration, invasion, and the regulation of antitumor immune systems. On the basis of these basic studies, new anticancer agents have been developed, and the efficacies and safeties of the new anticancer agents have been assessed by clinical trials. This Special Issue brings together original/review articles on basic and clinical research for sarcomas.

Recent development of molecular genetic techniques, including next-generation sequencing, has furthered understanding of the molecular pathogenesis of sarcomas. Choi et al. reviewed recent molecular findings and novel immunohistochemical markers in soft tissue tumors [1]. In their review article, molecular genetic alterations and immunohistochemical markers in various types of soft tissue tumors are summarized. These alterations and markers are thought to be diagnostic and therapeutic targets in soft tissue tumors.

Osteosarcoma is a highly aggressive bone sarcoma commonly affecting pediatric or adolescent patients, as well as large-breed dogs. Nance et al. applied single-nuclei multiome sequencing, encompassing ATAC (Assay for Transposase-Accessible Chromatin) and GEX (Gene Expression) sequencing, to canine osteosarcoma [2]. The comprehensive approach demonstrated the complexity of the tumor microenvironment by simultaneously capturing the transcriptomic and epigenomic profiles within the same nucleus. Furthermore, these results were analyzed in conjunction with bulk RNA sequencing and differential analysis of the same tumor- and patient-matched normal bone. In the study, significant heterogeneity in a single individual’s tumor was demonstrated.

The chimeric EWSR1::FLI1 transcription factor is widely known to be main oncogenic event in Ewing’s sarcoma. It has been reported that EWSR1::FLI1 levels are correlated with migratory and invasive phenotype. Fernández-Tabanera et al. investigated mechanisms of overexpression of CD44, a transmembrane protein involved in migration and adhesion, in the low EWSR1::FLI1 phenotypes [3]. In their study, expression of the CD44 inhibited cell proliferation in three Ewing’s sarcoma cell lines, and CD44 increased the cell migration of Ewing’s sarcoma cells. High-molecular-weight hyaluronic acid, a main ligand of CD44, blocked cell migration, while low-molecular-weight hyaluronic acid increased cell migration. Based on these findings, it is suggested that CD44s could be involved in the migration/invasion features that characterize the EWSR1::FLI1low phenotype.

In this Special Issue, there are two articles on promising therapeutic approaches for soft tissue sarcomas. Myxofibrosarcoma is a subtype of soft-tissue sarcoma, which is characterized by large intra-tumor heterogeneity and infiltrative growth patterns. The standard treatment of myxofibrosarcoma is surgical resection with sufficient surgical margin, and the effect of chemotherapy is limited. Lohberger et al. investigated the antitumor activity of shikonin derivatives in myxofibrosarcoma model [4]. In their study, shikonin and β,β-dimethylacrylshikonin dose-dependently inhibited the viability of tumor cells and induced apoptosis. Furthermore, shikonin derivatives inhibited pSTAT3 and increased pAKT, pERK, pJNK, and pp38. The results suggest that shikonin derivatives are promising anticancer agents in the treatment in myxofibrosarcoma. On the other hand, it has been reported that a series of synthetic chalcones (indolyl-pyridinyl-propenones; IPP) induce non-apoptotic cell death. Honkisz-Orzechowska et al. investigated the effect of the most active compounds, MIPP (3-(2-Methyl-1-H-indol-3-yl)-1-(4-pyridinyl)-2-propen-1-one) and MOMIPP (3-(5-methoxy-2-methyl-1H-indol-3-yl)-1-(4-pyridinyl)-2-propen-1-one) on HT-1080 fibrosarcoma cells [5]. In their study, MIPP and MOMIPP dose-dependently inhibited the viability of HT1080 cells. Both compounds induced the apoptosis and production of reactive oxygen species. These compounds caused massive vacuolization in HT1080 cells, and increased expression of LC3-II and the presence of autophagosomes with a double membrane. Their data suggested a promising link between autophagy and apoptosis in fibrosarcoma.

This Special Issue includes two articles on rhabdomyosarcoma (RMS). RMS in adults is a rare and aggressive disease, and there is no standard chemotherapy regimen for advanced rhabdomyosarcoma. Alveolar RMS is associated with chromosomal translocations, which result in oncogenic fusion genes PAX3-FOXO1 or PAX7-FOXO1. It has been reported that the overexpression of transforming growth factor beta (TGF-β) promotes epithelial to mesenchymal transition (EMT) through the increased expression of transcription factor SNAIL. In a review article reported by Bhushan, different types of RMS and the impact of TGF-β in the tumor types were described [6]. Furthermore, current chemotherapy strategies, including vincristine, actinomycin D, cyclophosphamide (VAC), cabozantinib, bortezomib, vinorelbine, AZD 1775, cisplatin, and other anticancer agents, targeting the differentiation of tumor cells in alveolar RMS, were discussed. Hindi et al. conducted a retrospective study of BOMP-EPI (bleomycin, vincristine, methotrexate and cisplatin alternating with etoposide, cisplatin and ifosfamide), an alternative platinum-based regimen, in adult patients with relapsed/metastatic RMS [7]. Among 10 study patients, 1 (10%) had complete response, 5 (50%) had partial response, 3 (30%) had stable disease, and 1 (10%) had progression of the disease. The median progression-free survival was 8.5 months and 7 patients died, with a median overall survival of 24.7 months. Based on the results, the platinum-based regimen seems to be active in adult patients with relapsed and advanced RMS. Further studies are necessary to investigate the efficacy and safety of the regimen in a large number of patients with advanced RMS.

Therapeutic options for bone and soft tissue sarcomas are limited, and clinical benefits from targeting therapies can only be seen in some of the patients. The rarity of tumors and the paucity of actionable mutations obstruct the development of new systemic treatments for bone and soft tissue sarcomas. Mancarella et al. reviewed targeted protein degradation (TPD), an innovative pharmacological modality, to alter protein abundance, based on the use of small molecules called degraders or proteolysis-targeting chimeras (PROTACs) [8]. In the review, features of PROTAC and PROTAC-derived genetic systems were discussed, as well as the potential of these approaches to overcome various problems of targeted therapies in sarcomas, including drug resistance, target specificity, and undruggable targets.

This Special Issue of the *International Journal of Molecular Sciences* has collected the latest original/review articles on bone and soft tissue sarcomas. Although current therapeutic options for bone and soft tissue sarcomas are limited, articles in this Special Issue have demonstrated appropriate treatment options, promising therapeutic targets, and new anticancer agents. We greatly appreciate the contribution of all of the authors of the articles published in this Special Issue.
